# 5-Fluorouracil induces apoptosis in rat cardiocytes through intracellular oxidative stress

**DOI:** 10.1186/1756-9966-31-60

**Published:** 2012-07-19

**Authors:** Monica Lamberti, Stefania Porto, Monica Marra, Silvia Zappavigna, Anna Grimaldi, Daniela Feola, Delia Pesce, Silvio Naviglio, Annamaria Spina, Nicola Sannolo, Michele Caraglia

**Affiliations:** 1 Occupational Medicine, Hygiene and Industrial Toxicology Section, Department of Experimental Medicine, Naples, Italy; 2 Department of Biochemistry and Biophysics, Second University of Naples, Via S.M. Costantinopoli, 16, 80138, Naples, Italy

**Keywords:** 5-Fluorouracil, Doxorubicin, Apoptosis, ROS, Cardiotoxicity, Health workers

## Abstract

**Background:**

Cardiotoxicity is a major complication of anticancer drugs, including anthracyclines and 5-fluorouracil(5-FU) and it can have detrimental effects both in patients and workers involved in the preparation of chemotherapy.

**Methods:**

Specifically, we have assessed the effects of increasing concentrations of 5-FU and doxorubicin (DOXO) on proliferation of H9c2 rat cardiocytes and HT-29 human colon adenocarcinoma cells by MTT assay. Cells were treated for 24, 48 and 72 h with different concentrations of the two drugs alone or with 5-FU in combination with 10^-4^ M of levofolene (LF).

**Results:**

5-FU induced a time- and dose-dependent growth inhibition in both cell lines. The 50% growth inhibition (IC:50) was reached at 72 h with concentrations of 4 μM and 400 μM on HT-29 and H9c2, respectively. The addition of LF to 5-FU enhanced this effect. On the other hand, the IC:50 of DOXO was reached at 72 h with concentrations of 0.118 μM on H9c2 and of 0.31 μM for HT-29. We have evaluated the cell death mechanism induced by 50% growth inhibitory concentrations of 5-FU or DOXO in cardiocytes and colon cancer cells. We have found that the treatment with 400 μM 5-FU induced apoptosis in 32% of H9c2 cells. This effect was increased by the addition of LF to 5-FU (38% of apoptotic cells). Apoptosis occurred in only about 10% of HT-29 cells treated with either 5-FU or 5-FU and LF in combination. DOXO induced poor effects on apoptosis of both H9c2 and HT-29 cells (5–7% apoptotic cells, respectively). The apoptosis induced by 5-FU and LF in cardiocytes was paralleled by the activation of caspases 3, 9 and 7 and by the intracellular increase of O^2−^ levels.

**Conclusions:**

These results suggest that cardiotoxic mechanism of chemotherapy agents are different and this disclose a new scenario for prevention of this complication.

## Introduction

Chemotherapy agents have a low therapeutic index thus affecting also normal cells and not only cancer counterparts. On this light, they induce often side effects in cancer patients that severely limit their activity. Moreover, many studies have assessed the risk of workers who handle anti-neoplastic drugs [1-15]. The health hazard for medical personnel administering these drugs is a major concern as these drugs are classified as potentially carcinogenic, mutagenic or teratogenic [16]. Exposure can occur mainly to hands and sporadically to other body parts as well. As these drugs directly or indirectly affect DNA, not only the cancer patients but also the medical personnel chronically handling these drugs are at a higher risk for acquiring DNA damage.

Cardiotoxicity is a major complication of anticancer drugs, including anthracyclines and 5-fluorouracil (5FU) [17-20]. Anthracyclines are the best studied among the anticancer drugs with established cardiotoxicity [21,22]. They produce cardiac toxicity accompanied by an increase in myofibrillar disarray that is mediated by the signaling function of neuregulin 1 [23]. In addition, anthracyclines induce mitochondrial apoptosis pathways and free radical production [24,25].

The mechanisms by which other chemotherapy drugs produce cardiovascular toxicities have also been investigated. 5-FU, a widely used chemotherapeutic, has direct toxic effects on vascular endothelium that involves endothelial nitric oxide (NO) synthase and leads to coronary spasms and endothelium-independent vasoconstriction via protein kinase C [26-32]. Therefore, also for this latter drug unexpected cardiotoxicity can occur above all in old patients who have often associated co-morbidities and can be defined frail patients. Above all in this latter category of patients, the understanding of the molecular mechanisms at the basis of the cardiotoxic effects induced by anti-cancer agents could be useful in order to determine possible pharmacological strategies in order to prevent this deleterious side effect. Moreover, the toxic effects on normal cells (cardiocytes) could differ from those induced in cancer cells (i.e.: colon cancer cells) and this could allow the use of cardioprotective agents without affecting the anti-cancer properties of 5-FU. It has also to be considered that an unexpected high risk of exposure to 5-FU was recently found in a population of workers of South Italy involved in the manipulation of cytostatic agents [33]. In the present study, we have evaluated the cardiotoxic effects of 5-FU and DOXO on rat cardiocytes (H9c2) [30] and a human colon adenocarcinoma (HT-29) cell line, already reported to be sensitive to 5-FU, for the study of the cell death pathways induced in cardiac and colon cancer cells.

## Materials and methods

### Materials

RPMI, DMEM, and FBS were purchased from Flow Laboratories (Milan, Italy). Tissue culture plasticware was from Microtech (Naples, Italy). Rabbit antiserum raised against caspase 9 and monoclonal antibodies (mAb) raised against caspase 3 and caspase 7 were purchased from Enzo Life Sciences (Florence, Italy). Hydroethidine conjugated secondary antibody was purchased from Sigma-Aldrich (Italy). Mouse antiserum raised against α−tubulin was purchased by Calbiochem (Merck KGaA, Darmstadt, Germany). 5-FU, Doxorubicin and were Levofolene were a gift of Dr. Gaetano Facchini (I.N.T. ‘Pascale’, Naples, Italy).

### Cell culture and proliferation

The rat cardiocytes (H9c2) cell line and the human colon adenocarcinoma (HT-29) cell line obtained from the American Type Tissue Culture Collection, Rockville, MD, grow in DMEM and RPMI1640, respectively, supplemented with heat inactivated 20% FBS, 20 mM HEPES, 100 U/ml penicillin, 100 mg/ml streptomycin, 1% L-glutamine and 1% sodium pyruvate. Both cell lines were grown in a humidified atmosphere of 95% air/5% CO_2_ at 37 °C.

Proliferation of H9c2 and HT-29 cell lines was performed in the presence of 5-FU and Doxorubicin (DOXO) in presence or not of Levofolene (LF), by MTT assay as previously described [28].

### Western blot analysis

H9c2 and HT-29 cell lines were grown for 48 h with or without DOXO or 5-FU in presence or not of LF at 37°C. For cell extract preparation, the cells were washed twice with ice-cold PBS, scraped and centrifuged for 30 min at 4°C in 1 ml of lysis buffer (1% Triton, 0.5% sodium deoxycholate, 0.1 NaCl, 1 mM EDTA, pH 7.5, 10 mM Na_2_HPO_4_, pH 7.4, 10 mM PMSF, 25 mM benzamidin, 1 mM leupeptin, 0.025 units/ml aprotinin). Equal amounts of cell proteins were separated by SDS-PAGE, electrotransferred to nitrocellulose and reacted with the different antibodies. Blot were then developed using enhanced chemiluminescence detection reagents (SuperSignal West Pico, Pierce) and exposed to x-ray film. All films were scanned by using Quantity One software (BioRad laboratories, Hercules, CA).

### Flow cytometric analysis of apoptosis

Annexin V-FITC (fluorescein isothiocyanate) was used in conjunction with a vital dye, Propidium Iodide (PI), to distinguish apoptotic (Annexin V-FITC positive, PI negative) from necrotic (Annexin V-FITC positive, propidium iodide positive) cells. Briefly, cells were incubated with Annexin-V–FITC (MedSystems Diagnostics, Vienna, Austria) and propidium iodide (Sigma, St. Louis, MO, USA) in a binding buffer (10 mM Hepes, pH 7.4, 150 mM NaCl, 5 mM KCl, 1 mM MgCl_2_, 2.5 mM CaCl_2_) for 10 min at room temperature, washed and resuspended in the same buffer. Analysis of apoptotic cells was performed by flow cytometry (FACScan, Becton Dickinson). For each sample, 2 × 10^4^ events were acquired. Analysis was carried out by triplicate determination on at least three separate experiments.

### Flow cytometric analysis of oxidative stress

The cells were seeded in 6-multiwell plates at the density of 3 × 10^5^ cells/well. After 24 h incubation at 37 °C the cells were treated for different time with the IC_50_s of 5-FU and DOXO. The oxidative stress was analysed by Hydroethidine (HE) staining after 48 h of treatment. Hydroethidine is used as a vital dye in fluorescence assays that operates as a probe for measurement of O_2_^−^. The dye enters the cells freely and is dehydrogenated to fluorescent ethidium bromide by O_2_^−^. Briefly, the cells were incubated for 1 h at the end of treatment with 20 ng/ml Hydroethidine stock solution (2,5 mg/ml). At the time of processing the cells were scraped, washed twice with PBS and the pellet was resuspended in 1 ml PBS. The dye accumulation was analysed by FACScan flow cytometer (FACScan, Becton Dickinson) by the CellQuest software. For each sample, 2 × 10^4^ events were acquired. Analysis was carried out by triplicate determination on at least three separate experiments.

### Statistical analysis

All data are expressed as mean + SD. Statistical analysis was performed by analysis of variance (ANOVA) with Neumann-Keul's multiple comparison test or Kolmogorov-Smirnov where appropriate.

## Results

### Effects of DOXO and 5-FU on H9c2 and HT-29 cell proliferation and apoptosis

**Figure 1:**
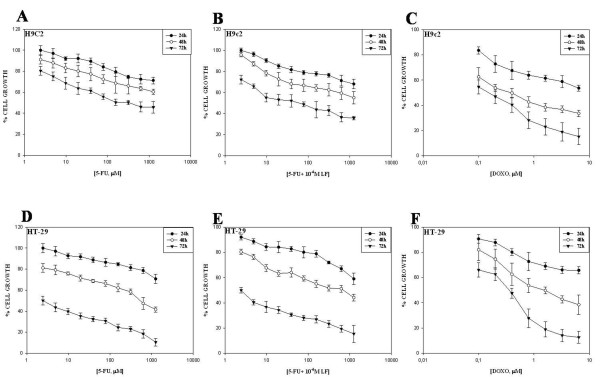
**Effects of DOXO and 5-FU on H9c2 and HT-29 cell proliferation.** Growth inhibition of H9c2 (**A**-**C**) and HT-29 (**D**-**F**) cells treated with 5-FU alone (**A** and **D**) or combined with LF (**B** and **E**) or DOXO alone (**C** and **F**) for 24, 48 and 72 h, evaluated by MTT assay and expressed as a percentage of untreated cells. Data are reported as mean of three independent experiments ± SD. The experiments were repeated at least three times and gave always similar results.

We studied the effect of increasing concentrations of DOXO and 5-FU in presence or not of LF on growth inhibition of HT-29 and H9c2 cells by MTT assay as described in “Materials and Methods”. We have found a dose and time-dependent growth inhibition in both cell lines. In details, the IC_50_ (50% inhibitory concentration) value of 5-FU was 4 μM and 400 μM in HT29 and H9c2, respectively (Figure 1 and Table 1). Moreover, LF potentiated growth inhibition induced by 5-FU. In fact, IC_50_ of HT-29 and H9c2 cells was 2 μM and 43 μM, respectively. These results suggest, as expected, that the colon cancer cell line HT29 was more sensitive to 5-FU than H9c2 normal cells (Table 1). Interestingly, these concentrations of 5-FU can be reached in vivo after the routinely used ways of administration of this agent in the clinical practice [34].

**Table 1:** **IC**_**50**_**s of the different drugs in cardiocytes and colon cancer cells**

***Drugs***	***IC***_***50***_***H9c2***	***IC***_***50***_***HT-29***
*5-FU*	400 μM ± 0.06	4 μM ± 0.01
*5-FU* + *10*^*−4*^*M LF*	43 μM ± 0.01	2 μM ± 0.009
*DOXO*	0.12 μM ± 0.001	0.31 μM ± 0.002

On the other hand, H9c2 cells appeared to be more sensitive to DOXO than HT-29. In fact, the IC_50_ of DOXO was 0.12 μM and 0.31 μM on HT-29 and H9c2, respectively (Figure 1).

**Figure 2:**
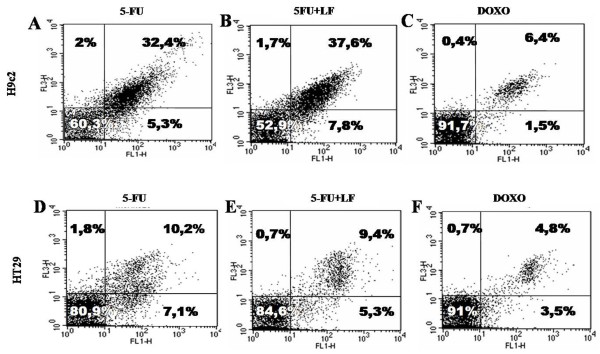
**Effects of DOXO and 5-FU on H9c2 and HT-29 apoptosis.** FACS analysis after double labelling with PI and Annexin V of H9c2 (**A**–**C**) and HT-29 (**D**–**F**) cells treated with 5-FU alone (**A** and **D**) or combined with LF (**B** and **E**) or DOXO alone (**C** and **F**). The experiments were performed at least three times and the results were always similar. Insets, % of positive cells.

Thereafter, we have evaluated the effects of the different treatments in inducing apoptosis, assessed by FACS analysis after double labelling with Annexin V and PI. We have found that the treatment with DOXO induced apoptosis in only about 8% of H9c2 cell population (Figure 2 and Table 2), while the treatment with 5-FU alone induced apoptosis in about 38% of H9c2 cell population compared to 5% of untreated cells as demonstrated with FACS analysis. Moreover, when the cells were treated with 5-FU and exposed to LF about 45% apoptosis was found. Only about 17% and 8% apoptosis was induced by DOXO and 5-FU, respectively in HT-29 cell line (Figure 2 and Table 2). Therefore, DOXO and 5-FU caused antiproliferative effects in cardiocytes and tumour cells with different mechanisms.

**Table 2:** Study of apoptosis in H9c2 and HT-29 cell line

**72 h H9c2**	**Necrosis**	**Late apoptosis**	**Alive**	**Early apoptosis**
CTR	0.11	1.11	98.4	0.38
5-FU	2.09	32.36*	60.25	**5.30**
LF	0.19	0.06	99.73	0.02
5-FU + LF	1.7	**37.6**	52.9	**7.75**
DOXO	0.43	6.35	91.69	1.53
**72 h HT29**	**Necrosis**	**Late apoptosis**	**Alive**	**Early apoptosis**
CTR	0.16	0.01	99.66	0.17
5-FU	1.84	**10.15**	80.86	**7.15**
LF	1.93	0.48	97.21	0.38
5-FU + LF	0.68	**9.39**	84.63	**5.30**
DOXO	0.67	**4.8**	90.98	**3.55**

* In bold: significant changes.

### Modulation of intracellular levels of ROS

To evaluate the intracellular levels of ROS, HT-29 and H9c2 cells were incubated with dihydroethidine followed by FACS analysis of the oxidative product, ethidium, which emits red fluorescence. The mean fluorescence intensity (MFI) corresponds to ROS levels and to intracellular oxidative stress due to superoxide anion (O^2−^) generation induced by their presence.

**Figure 3:**
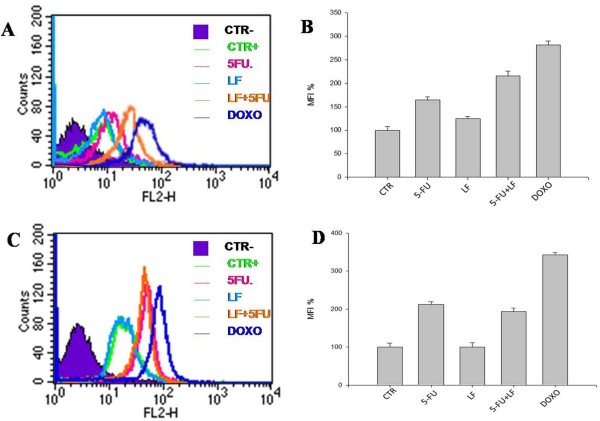
**Modulation of intracellular levels of ROS.** H9c2 and HT-29 were incubated with dihydroethidine and analyzed by flow cytometry as described in “Materials and Methods”. (**A**,**C**) Flow cytometric analysis of H9c2 (**A**) and HT-29 (**C**) cells treated with 5-FU alone or combined with LF or DOXO alone exposed to dihydroethidine used as a probe for measurement of O_2_^−^. (**B**,**D**) Representation of the ROS levels expressed as the percentage of mean fluorescence intensity (MFI) derived by dihydroethidine oxidation of H9c2 (**B**) and HT-29 (**D**) cells treated with 5-FU alone or combined with LF or DOXO alone. The experiments were repeated at least three times and gave always similar results. Bars, SDs.

In H9c2 cells, 5-FU caused an about 1.5-fold increase of MFI reaching an increase of about 2-fold of MFI with the addition of LF indicating a potentiation of oxidative effects (Figure 3 A,B). In the same experimental conditions, we observed an about 3-fold increase of MFI induced by DOXO treatment. In HT29 cells, LF did not potentiate the increase of MFI induced by 5-FU alone that was of about 2-fold while DOXO induced an about 3-fold increase of MFI. Therefore, the oxidative stress induced by DOXO was more potent than that one caused by 5-FU in both cancer cells and cardiocytes. Moreover, LF potentiated the oxidative stress induced by 5-FU only in cardiocytes and not in colon cancer cells.

### Effects of the different treatments on caspase activation

In order to characterize the apoptosis induced in these cell lines we have evaluated if the different treatments induced the activation of mediators of the execution phase of apoptosis.

**Figure 4:**
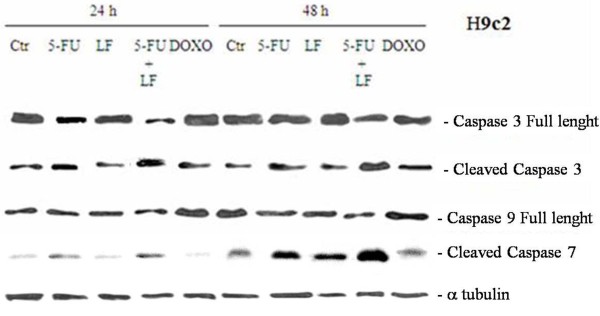
**Effects of the different treatments on caspase activation in H9c2 cells.** H9c2 cells were treated with 5-FU alone or combined with LF or DOXO alone for 48 h at the concentrations inhibiting the 50% of the proliferation of the cardiocytes as previously indicated in Table 1. Thereafter, the expression of caspase 3, 7 and 9 were evaluated after blotting with specific antibodies that recognise both the full and the cleaved forms of the proteins, as described in "Materials and Methods". Expression of the house-keeping protein α-tubulin, used as loading control, was also evaluated. The experiments were performed at least three different times and the results were always similar. CTR, untreated cells; 5-FU, cells treated with 5-FU alone; 5-FU + LF, cells treated with 5-FU in combination with LF; DOXO, cells treated with DOXO alone.

**Figure 5:**
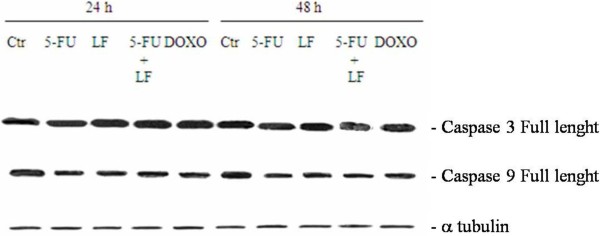
**Effects of the different treatments on caspase activation in HT29 cells.** HT-29 cells were treated with 5-FU alone or combined with LF or DOXO alone for 48 h at the concentrations inhibiting the 50% of the proliferation of the colon cancer cells as previously indicated in Table 1. Thereafter, the expression of caspase 3 and 7 were evaluated after blotting with specific antibodies that recognise the full form of the proteins, as described in "Materials and Methods". Expression of the house-keeping protein α-tubulin, used as loading control, was also evaluated. The experiments were performed at least three different times and the results were always similar. CTR, untreated cells; 5-FU, cells treated with 5-FU alone; 5-FU + LF, cells treated with 5-FU in combination with LF; DOXO, cells treated with DOXO alone.

We have evaluated the cleavage and the consequent activation of both caspase 9 and 3 with western blotting using specific antibodies that recognize only the intact forms of the two enzymes. We have found that 5-FU increased the cleavage of caspase 3 in H9c2 cells and the latter was potentiated in presence of LF. These effects were paralleled by a decrease of pro-caspase 9 expression (activation index). On the other hand, DOXO increased the cleavage of caspase 3 and 9 after 24 h from the beginning of treatment but the latter returned to basal level after 48 h (Figure 4). Moreover, the different treatments caused no significant changes of the levels of pro-caspase 3 and 9 in HT29 cell line (Figure 5).

These results were consistent with the data derived from FACS analysis; in fact, the treatment with 5-FU and LF induced a stronger apoptotic effect on cardiocytes cell line if compared with that one recorded in colon adenocarcinoma cell line.

### Intracellular increase of ROS is responsible for apoptosis induced by 5-FU in cardiocytes

**Figure 6:**
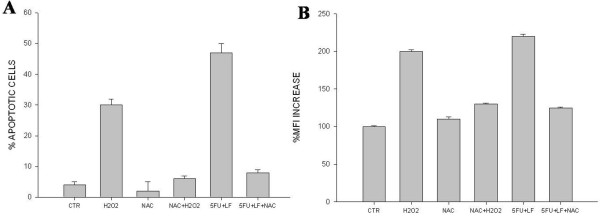
**Effects of the scavenger NAC on both oxidative stress and apoptosis of H9c2cells.A**) FACS analysis after double labelling with PI and FITC-Annexin V of H9c2 cells treated with 5-FU combined with LF or 200 μμ H_2_O_2_ or NAC alone or in combination for 48 h. The experiments were performed at least three times and the results were always similar. The results show the % of apoptotic cells derived from the sum of the events calculated as late and early apoptotic cells. Bars, SEs. CTR, untreated cells; H_2_O_2_, cells treated with 200μM H_2_O_2_ alone; NAC, cells treated with 5 mM NAC alone; NAC + H_2_O_2,_ cells treated with 5 mM NAC +200μM H_2_O_2_; 5-FU + LF, cells treated with 5-FU in combination with LF; 5-FU + LF + NAC, cells treated with 5-FU in combination with LF and 5 mM NAC. **B**) H9c2 were incubated with dihydroethidine and analyzed by flow cytometry as described in “Materials and Methods”. Flow cytometric analysis of H9c2 cells treated with 5-FU combined with LF or 200 μM H_2_O_2_ or NAC alone or in combination exposed to dihydroethidine used as a probe for measurement of O_2_^−^. Representation of the ROS levels expressed as the percentage of mean fluorescence intensity (MFI) derived by dihydroethidine oxidation of H9c2 cells. The experiments were repeated at least three times and gave always similar results. Bars, SDs. CTR, untreated cells; H_2_O_2_, cells treated with 200μM H_2_O_2_ alone; NAC, cells treated with 5 mM NAC alone; NAC + H_2_O_2,_ cells treated with 5 mM NAC +200μM H_2_O_2_; 5-FU + LF, cells treated with 5-FU in combination with LF; 5-FU + LF + NAC, cells treated with 5-FU in combination with LF and 5 mM NAC.

In order to study the role of the oxidative stress in the induction of apoptosis by 5-FU in cardiocytes we have exposed H9c2 to the scavenger agent N-acetyl cysteine (NAC) and we have evaluated the effects on apoptosis and intracellular ROS. The addition of NAC alone to H9c2 cells had no effects on apoptosis and intracellular ROS (Figure 6A, B respectively). On the other hand, the addition of NAC to either 5-FU alone or in combination with LF completely abrogated the effects of both on apoptosis and increase in the levels of ROS (Figure 6A, B respectively). We have also used H_2_O_2_ as positive control and we have found that the addition of 200 μM H_2_O_2_ to H9c2 cells caused an about 40% apoptosis with an about 2-fold increase of intracellular ROS and that these effects were again abrogated by the concomitant administration of NAC (Figure 6A, B respectively).

These results strongly suggested that apoptosis induced by 5-FU in cardiocytes is likely due to the increase in intracellular ROS.

## Discussion

In this study we have compared the effects induced by either 5-FU ± LF or DOXO on proliferation of both cardiocytes H9c2 cell line and human colon adenocarcinoma HT-29 cells. We have found that the antiproliferative activity of 5-FU ± LF was more pronounced in colon cancer cells than on cardiocytes and this effect was not surprising since this was on line with previous data demonstrating the in vitro activity of these drugs in colon cancer cell lines [35,36]. Thereafter, we have characterized the mechanism of cell death caused by the different treatments and we have found that cardiocytes were more sensitive to apoptosis induced by 5-FU and LF than colon cancer cells. In other words, the cytotoxicity recorded in cardiocytes was in the most part due to the induction of apoptosis while that one determined in colon cancer cells was due to a different mechanism (likely necrosis or autophagy or both). These results are not surprising on the basis of the reported side effects of 5-FU. In fact, typical side effects of 5-FU are myelosupression, nausea, vomiting, diarrhea and stomatitis [37]. Cardiotoxicity is the other toxicity [36]. Cardiac side effects are ST segment changes, rhythm abnormalities, supraventricular and ventricular dysrhythmias [38] and acute myocardial infarction was also reported in the literature [39]. In fact, cardiocytes have protective mechanisms that overcome the apoptotic injury caused by several toxic agents that can circulate in the bloodstream among which cytotoxic drugs as in the case of cancer patients treated with chemotherapy [40]. Unfortunately, this program is not able to avoid the injury induced by agents with a very high oxidative potential as some anti-cancer agents. Moreover, cardiocytes are more prone to go towards the apoptotic program because, differently from cancer cells, have a poor amplification of the protective anti-apoptotic pathways. The latter are essential in order to allow the development and spreading of cancer cells into the whole organism and cancer cells have the opportunity to develop them during their long natural history [41].

On the other hand, the increase of the intracellular ROS caused by 5-FU ± LF on both H9c2 and HT-29 was less than that one determined by DOXO and this effect was likely due to the reported sensitivity of heart to the oxidative stress induced by DOXO. Several mechanisms of the intracellular oxidative stress have been reported, including generation of free radicals and lipid peroxidation of cardiac membranes [3], myocyte damage induced by cardiac calcium overload [4], formation of DOX-iron complex [5], impaired myocardial adrenergic regulation, cellular toxicity of anthracycline metabolites [6], and inhibition of beta-oxidation of long chain fatty acids with the consequent depletion of cardiac ATP [7]. The study of the activation of caspase cascade suggested a mytochondria-mediated triggering of the apoptotic program in cardiocytes that is conceivable with the involvement of oxidative stress. In order to definitively study the relevance of the increase of intracellular ROS in the induction of apoptosis induced by 5-FU ± LF, we have treated cardiocytes with the scavenger NAC and we have studied the effects on the apoptosis occurrence [42]. We have indeed found that the addition of NAC to the 5-FU ± LF-treated cardiocytes was able to completely antagonize the apoptosis.

In conclusion, our data suggest that agents such as 5-FU different from anthracyclines, that are conventionally related to the induction of cardiotoxic effects, can also induce cardiocyte damage through the triggering of an apoptotic program. This detrimental effect could be due to the induction of the increase of intracellular ROS and strategies based upon the use of scavengers such as NAC could be used in order to prevent this effect. The data obtained in this study will be confirmed in vivo with a series of experiments already in preparation.

## Abbreviations

5-FU, 5-fluorouracil; DOXO, Doxorubicin; LF, Levofolene; NO, Nitric oxide; H9c2, Rat cardiomyocytes; HT-29, Human colon adenocarcinoma cell line; HE, Hydroethidine; MFI, Mean fluorescence intensity; NAC, N-acetyl cysteine.

## Competing interests

The authors declare that they have no competing interests.

## Authors’ contributions

ML and MC carried out the design of the experiments and drafted the manuscript. SP, SZ, AG, DF and DP participated in the experiments of cell culture and molecular biology. MM participated in statistical analysis and interpretation. SN, NS and AS participated in the design of the experiments. All authors read and approved the final manuscript.
